# Gastric MALT Lymphoma with Increased Plasma Cell Differentiation Showing Unique Endoscopic Features

**DOI:** 10.1155/2018/8054284

**Published:** 2018-02-14

**Authors:** Masaya Iwamuro, Takehiro Tanaka, Kenji Nishida, Seiji Kawano, Yoshiro Kawahara, Shogen Ohya, Tadashi Yoshino, Hiroyuki Okada

**Affiliations:** ^1^Department of Gastroenterology and Hepatology, Okayama University Graduate School of Medicine, Dentistry, and Pharmaceutical Sciences, Okayama 700-8558, Japan; ^2^Department of Pathology, Okayama University Hospital, Okayama 700-8558, Japan; ^3^Department of Pathology, Okayama University Graduate School of Medicine, Dentistry, and Pharmaceutical Sciences, Okayama 700-8558, Japan; ^4^Department of Endoscopy, Okayama University Hospital, Okayama 700-8558, Japan; ^5^Kawaguchi Medical Clinic, Okayama 700-0913, Japan

## Abstract

A 62-year-old woman was diagnosed with extranodal marginal zone lymphoma of mucosa-associated lymphoid tissue (MALT lymphoma) with increased plasma cell differentiation of the stomach. Esophagogastroduodenoscopy showed slightly elevated, whitish lesions in the gastric body. Magnifying endoscopic observation revealed that the gastric surface epithelium was swollen, but the structure was not destroyed or diminished. Elongated, tortuous vasculature was observed on the surface of the whitish lesions. The patient underwent eradication treatment for* Helicobacter pylori*, which resulted in complete remission. Although the appearance of abnormal vessels and the destruction of gastric epithelial structure are the typical features of gastric MALT lymphoma during magnifying endoscopy, the present case showed different features, which were rather similar to those observed in a previously reported case of gastric plasmacytoma. The current case indicates that magnifying endoscopic features are not uniform among gastric MALT lymphomas.

## 1. Introduction

Extranodal marginal zone lymphoma or mucosa-associated lymphoid tissue (MALT lymphoma) is one of the most common non-Hodgkin lymphomas arising in the gastrointestinal tract, particularly in the stomach [1]. Gastric MALT lymphomas exhibit various types of morphologies, ranging from erosions/ulcers, early gastric cancer-like lesions, whitish mucosa, and cobblestone appearance to submucosal tumor [2]. Magnifying endoscopic observation of gastric MALT lymphomas shows typical features such as the appearance of abnormal vessels and the destruction of gastric epithelial structure [3–5]. Since these features disappear when pathological remission is achieved [5], understanding these features is essential for proper detection and management of gastric MALT lymphomas.

Recently, we encountered a case of gastric MALT lymphoma with increased plasma cell differentiation. The gastric lesions exhibited unique endoscopic features, showing slightly elevated, whitish lesions, with a swollen epithelium but intact epithelial structure. Elongated, tortuous vasculature was observed on the surface of the whitish lesions, suggesting the deposition of whitish substances beneath the gastric epithelium. These features identified in magnifying endoscopic observation were similar to those observed in a previously reported case of gastric plasmacytoma [6]. In this report, we focus mainly on the pathologic and endoscopic features of our patient.

## 2. Case Report

**Figure 1:**
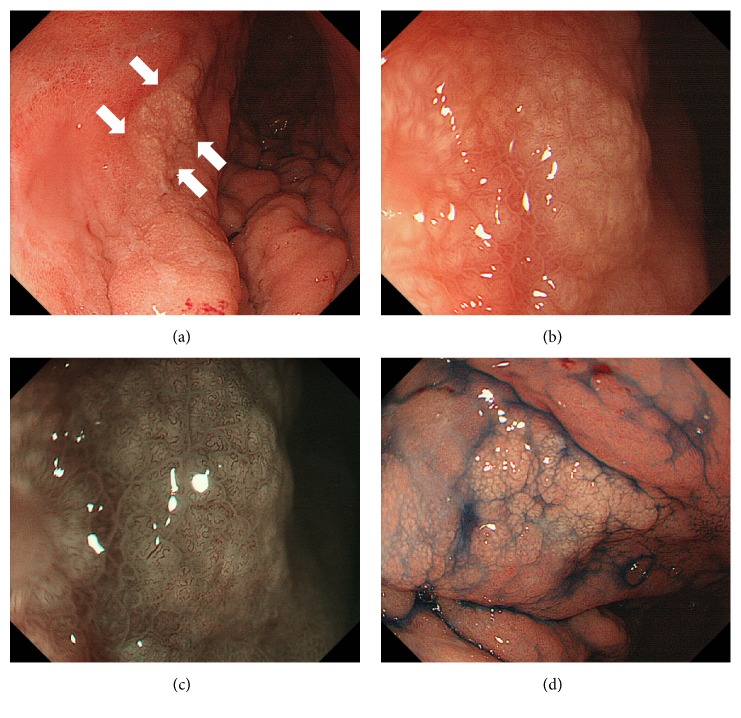
Esophagogastroduodenoscopy images. Slightly elevated, whitish lesions are observed in the gastric body ((a) arrows). Magnifying endoscopic observation with white light (b) and with narrow-band imaging (c) reveals that the gastric surface epithelium is swollen, but the structure is not destroyed or diminished. Whitish area is emphasized after indigo carmine spraying (d).

A 62-year-old woman tested positive for serum anti-*Helicobacter pylori* IgG antibody at her annual medical checkup. Subsequently, she underwent esophagogastroduodenoscopy at her family clinic, which revealed a whitish area in the gastric body. The patient was referred to Okayama University Hospital for further investigation and treatment. She had been taking rosuvastatin for hyperlipidemia and had no history of gastrointestinal diseases. A physical examination revealed no abnormalities, and there were no lymphadenopathies or hepatosplenomegaly. Laboratory findings including hemoglobin, lactate dehydrogenase, soluble interleukin-2 receptor, and immunoglobulin M, G, and A levels were within the normal ranges. There was no M protein in serum or urine protein electrophoresis. Monoclonal protein was not identified in the serum or urine by using immunoelectrophoresis. Esophagogastroduodenoscopy (GIF-H260Z; Olympus, Tokyo, Japan) showed slightly elevated, whitish lesions in the gastric body (Figure 1(a)). Magnifying endoscopic observation with white light (Figure 1(b)) and with narrow-band imaging (Figure 1(c)) revealed that the gastric surface epithelium was swollen, but the structure was not destroyed or diminished. Elongated, tortuous vasculature was observed on the surface of the whitish lesions, suggesting the deposition of whitish substances beneath the gastric epithelium.

**Figure 2:**
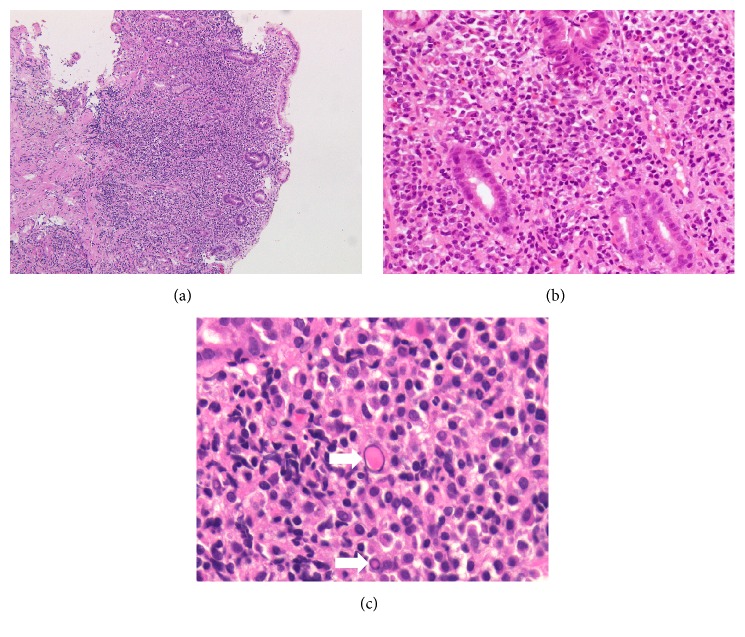
Pathological images. Hematoxylin and eosin staining shows dense, diffuse infiltration of small-to-medium-sized monocytoid cells and plasma cells (a, b) and multiple Dutcher bodies ((c) arrows).

**Figure 3:**
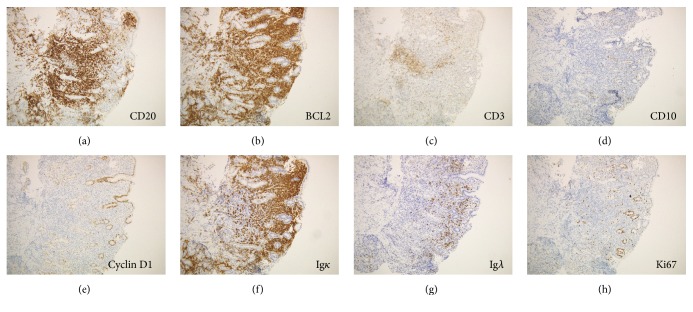
Pathological images with immunostaining. The infiltrating cells are positive for CD20 (a) and BCL2 (b), while they are negative for CD3 (c), CD10 (d), and cyclin D1 (e). In situ hybridization for immunoglobulin light chains shows positive results for immunoglobulin light chain *κ* (f) and negative results for light chain *λ* (g).

**Figure 4:**
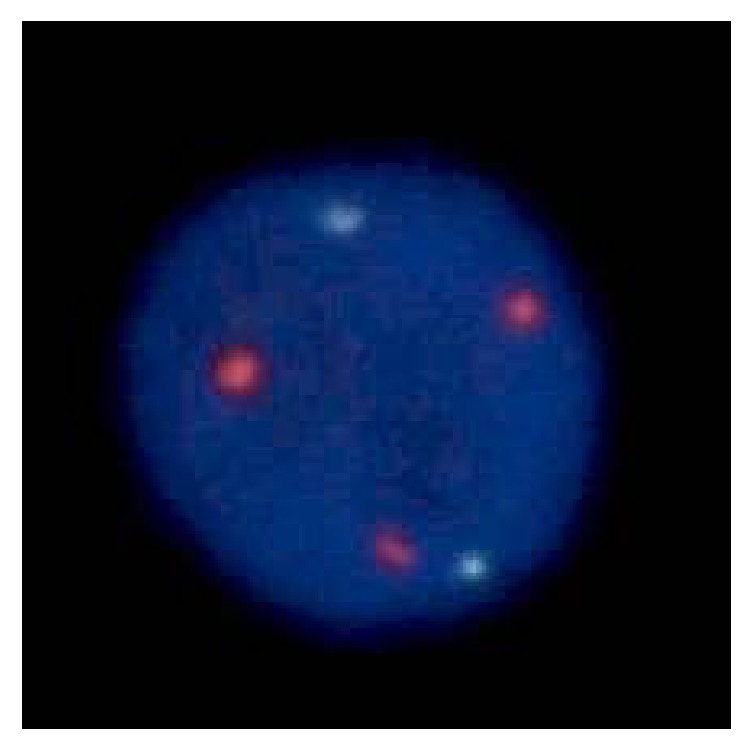
Fluorescence in situ hybridization image. Analysis for t(11;18)(q21;q21) translocation reveals no fusion genes of* BIRC3*-*MALT1*, although extra copies of* MALT1* are identified, indicating trisomy of chromosome 18.

Biopsy samples from the gastric lesions showed dense, diffuse infiltration of small-to-medium-sized monocytoid cells and plasma cells and multiple Dutcher bodies (Figure 2). Lymphoepithelial lesions were absent. Immunohistochemistry analysis showed that the infiltrating cells were positive for CD20 and BCL2, while they were negative for CD3, CD10, and cyclin D1 (Figure 3). In situ hybridization for immunoglobulin light chains showed expression of monoclonal immunoglobulin light chain *κ* in these cells (Figures 3(f) and 3(g)). Few tumor cells were positive for Ki-67 staining, indicating few mitotic cells. The diagnosis of gastric MALT lymphoma with plasma cell differentiation was made based on these pathological features. Fluorescence in situ hybridization (FISH) analysis for t(11;18)(q21;q21) translocation revealed no fusion genes of* BIRC3-MALT1*, although extra copies of* MALT1* were identified in 32.0% of the monocytoid cells (Figure 4), indicating trisomy of chromosome 18 [2, 7]. Chromosome banding of the bone marrow aspirate showed a normal karyotype of 46, XX, indicating no congenital chromosomal abnormalities.

Colonoscopy showed no infiltration of monocytoid B-cells. Bone marrow aspiration and a biopsy also showed no monocytoid B-cells. Contrast-enhanced computed tomography imaging of the neck, chest, abdomen, and pelvis demonstrated no lymph node enlargement or organ involvement. Positron emission tomography disclosed tracer uptake in the gastric body, but there was no uptake in other organs. Based on these findings, the patient was diagnosed with primary gastric MALT lymphoma exhibiting prominent plasma cell differentiation.

**Figure 5:**
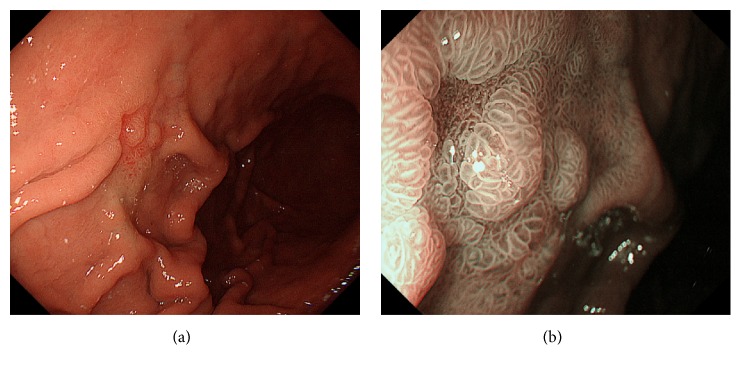
Esophagogastroduodenoscopy images after treatment. Three months after successful eradication of* H. pylori*, the whitish lesions disappeared.

Since the patient tested positive for* H. pylori* infection serologically and pathologically and as the urea breath test showed positive results, eradication of* H. pylori* was performed as a first-line therapy for gastric MALT lymphoma. Esophagogastroduodenoscopy performed three months after successful eradication of* H. pylori* revealed that the whitish lesions had disappeared (Figure 5). Remission was pathologically confirmed on the biopsied specimen.

## 3. Discussion

Plasma cell differentiation is not a rare event in MALT lymphoma [8]. This feature is reportedly more frequent in thyroid MALT lymphomas than in MALT lymphomas occurring in other organs [9]. Plasma cell differentiation occurs in approximately one-third of the cases of primary gastric MALT lymphomas [1, 10]. However, prominent plasma cell differentiation, as observed in the present case, is rarely observed in gastric MALT lymphomas.

Recently, Park et al. investigated the clinicopathological features of gastric MALT lymphoma with increased plasma cell differentiation [10]. The authors retrospectively compared 36 cases with increased plasma cell differentiation and 16 cases with minimal plasma cell differentiation. They reported that pathological response, that is, complete histologic response or probable minimal residual disease, was more frequently achieved after* H. pylori* eradication in gastric MALT lymphomas with increased plasma cell differentiation, compared with that in lymphomas with minimal plasma cell differentiation (94.4% versus 66.7%). Moreover, relapse was less frequent in cases with increased plasma cell differentiation (5.6% versus 35.7%). These results indicate that increased plasma cell differentiation is an indicator of favorable treatment response. The present patient also showed complete histologic response after successful* H. pylori* eradication.

**Figure 6:**
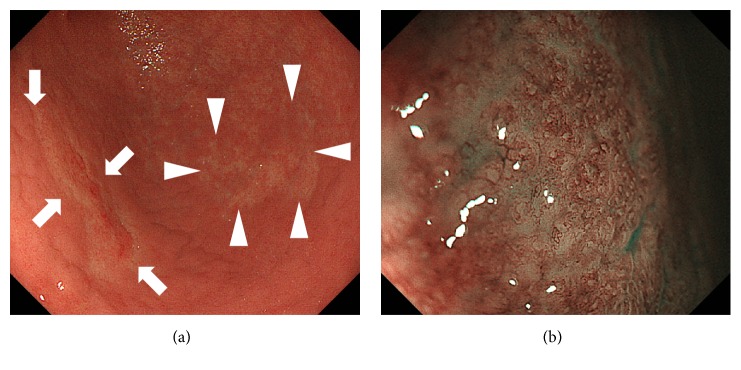
Endoscopic images of typical gastric MALT lymphoma. Two whitish lesions are observed in the gastric body ((a) arrows and arrowheads). Magnifying observation reveals branched vessels and fading of the gastric epithelial structure (b).

As described above, the appearance of abnormal vessels and the destruction of gastric epithelial structure have been known as the key magnifying endoscopic features of gastric MALT lymphomas [3–5].  Figure 6 shows the endoscopic images of typical gastric MALT lymphoma in a 46-year-old Japanese woman. There were two whitish lesions in the gastric body (Figure 6(a)), with branched vessels and faded gastric epithelial structure (Figure 6(b)), as observed in the magnifying observation. Ono et al. reported the disappearance of gastric pits and appearance of abnormal vessels in all 11 of their patients with gastric MALT lymphoma [3]. Moreover, after achieving a complete response of MALT lymphoma, abnormal vessels were no longer detected and gastric pits reemerged, although with an irregular size and formation pattern throughout the lesion; moreover, the subepithelial capillary network had unequal diameters. Subsequently, the same group investigated the magnifying endoscopic features of 21 patients with gastric MALT lymphoma and reported that nonstructural areas and abnormal vessels were positive in all lymphoma lesions (100%) before initiating treatment [5]. In addition, swelling of the crypt epithelium, which was termed as “ballooning,” was noted in 11 patients (52.4%). Nonaka et al. used the term “tree-like appearance,” which was defined as abnormal blood vessels resembling branches from the trunk of a tree, in which the gastric glandular structure was lost. The authors reported that they identified the tree-like appearance in 12 out of 16 patients with gastric MALT lymphoma (75.0%) during magnifying esophagogastroduodenoscopy observation with narrow-band imaging [4, 11, 12]. Consequently, unusually shaped vasculature and destruction of gastric pits with a nonstructural pattern appear to be representative features of untreated gastric MALT lymphomas.

In the present patient, although swelling of the gastric surface epithelium was observed, the structure of gastric pits was intact. Vasculature on the lesion showed an elongated, tortuous appearance, but it was not “tree-like.” Therefore, the magnifying endoscopic features of the present case were different from those of typical gastric MALT lymphomas. Nevertheless, we noticed several similarities between the magnifying endoscopic features of the present case and a case of gastric plasmacytoma reported by Harada et al. [6], in which the gastric lesion was described as a discolored, slightly elevated area with tortuous superficial vessels.

Extramedullary plasmacytoma is defined as an accumulation of neoplastic monoclonal plasma cells occurring in the extraosseous site without evidence of a systemic plasma cell proliferative disorder [13, 14]. Several authors have noted that the distinction between plasmacytoma and MALT lymphoma with plasma cell differentiation is sometimes ambiguous [8, 15]. Meanwhile, Meyerson et al. investigated both diseases and related disorders by using flow cytometric analysis and described that plasma cells observed in marginal zone lymphoma resembled normal precursor plasma cells, whereas those observed in plasma cell myeloma were similar to more mature marrow plasma cells [16]. Regardless of the pathogeneses of the two diseases, the similarities in the endoscopic images observed in the present case and the case of plasmacytoma reported by Harada et al. [6] may reflect common pathological features shared between the two cases, for example, proliferation of plasma cells. However, further studies are required to determine the macroscopic morphologies of gastric MALT lymphoma with increased plasma cell differentiation, as we had previously encountered another case of this disease, which showed a lack of gastric pits and the presence of abnormal vessels [7].

Another feature that may concern the outcome of the present case is extra copies of* MALT1*, which was observed in FISH analysis (Figure 4). Recently, we investigated 146 patients with gastric MALT lymphoma and found extra copies of* MALT1* in 31 patients (21.2%) and t(11;18) translocation in 27 patients (18.5%) [2]. Analysis of the patient outcome revealed that* H. pylori* eradication alone resulted in complete remission in 13 (61.9%) patients with extra copies of* MALT1*. The response rate to* H. pylori* eradication in this patient group was similar to that of patients without chromosomal aberrations (72.1%). However, although the difference was not statistically significant, event-free survival of the patients with extra copies of* MALT1* tended to be inferior to that of the patients without chromosomal aberration (*p* = 0.10). Therefore, we speculate that patients with additional copies of* MALT1*, including the present patient, may require more frequent clinical follow-ups to monitor disease progression and relapse.

In conclusion, this patient with gastric MALT lymphoma with increased plasma cell differentiation was treated. Although the gastric epithelium was swollen and elongated, tortuous vasculature was observed, branched microvessels were absent, and the gastric pits were preserved, under magnifying observation. These images were different from the typical features of gastric MALT lymphoma, but were similar to those of a previously reported case of gastric plasmacytoma. This case indicates that magnifying endoscopic features are not uniform among gastric MALT lymphomas and that increased plasma cells may be responsible for such atypical features.
